# Editorial: Merging symbolic and data-driven AI for robot autonomy

**DOI:** 10.3389/frobt.2025.1662674

**Published:** 2025-07-23

**Authors:** Daniele Meli, Mohan Sridharan, Simona Perri, Nikos Katzouris

**Affiliations:** ^1^Department of Computer Science, University of Verona, Verona, Italy; ^2^School of Informatics, University of Edinburgh, Edinburgh, United Kingdom; ^3^Department of Mathematics and Computer Science (DeMaCS), University of Calabria, Arcavacata di Rende, Italy; ^4^ National Centre of Scientific Research “Demokritos”, Agia Paraskevi, Greece

**Keywords:** neurosymbolic AI, probabilistic reasoning, reasoning under uncertainty, hybrid AI, robotics

Robots are increasingly being deployed to assist humans in many applications such as medicine, navigation, and industrial automation. To truly collaborate with humans in complex environments, robots require advanced cognitive capabilities, including the ability to reason with domain-specific commonsense knowledge and the noisy observations obtained in the presence of partial observability and non-deterministic action outcomes. Research in Artificial Intelligence (AI) has resulted in sophisticated symbolic formalisms based on logics to represent commonsense domain knowledge, as well as probabilistic and data-driven frameworks that quantitatively represent uncertainty in the decisions made by robots.

By themselves, symbolic or stochastic AI methods have limitations when applied to robots in complex scenarios. Symbolic AI methods reason with relational descriptions of the attributes of the domain and the robot to guide the robot’s behavior. At the same time, they tend to require extensive prior knowledge about the domain and the robot. They also make it computationally expensive to operate at the level of granularity required for precise interaction with the physical world, or to reason about uncertainty quantitatively. Probabilistic and data-driven AI methods, on the other hand, elegantly represent uncertainty quantitatively, and provide mechanisms for reasoning and acting at the level of granularity required for interaction with the physical world. These methods, however, offer limited expressiveness for complex cognitive concepts, and it is not always meaningful to reason about uncertainty quantitatively. With the increasing use of AI and robots in different applications, there has been renewed interest in hybrid and neurosymbolic AI frameworks that combine symbolic and data-driven methods. The 10 contributions in this Research Topic highlight the promise and potential of such frameworks in the context of robotics.

Describing a vision for the future, Spasokukotskiy states that the next-generation AI systems should not only be endowed with autonomy but also *“morality” that secures alignment in large systems*, i.e., they should operate safely within the values of human society. Instead of being in full control of AI, humans would then cooperate and communicate with intelligent systems. Extending this idea, Pal explains *the relevance of transparency, explainability, learning from a few examples, and the trustworthiness of an AI system*, exploring how insights into human reasoning can be a crucial ingredient for achieving reliable operation with embodied AI systems. In addition, Toberg et al. provide a systematic review of robot systems that represent, reason with, learn, and/or use commonsense knowledge in a wide range of application domains. Symbolic AI methods can play a crucial role in the design of such AI/robotics systems, providing the expressivity for elegantly representing human-level concepts and effectively modeling logical reasoning capabilities. These methods can also support more efficient and transparent learning, and the use of human guidance to generate symbolic abstractions. Das et al. describe a framework that extends an inductive logic program learner to demonstrate this capability on multiple benchmark domains, one of which focuses on planning the assembly of mechanical structures, a core task in industrial automation.

In addition to reasoning with prior knowledge that includes cognitive theories, robots that interact with the physical world process a large amount of continuous multi-modal inputs from different information sources, including humans and other agents. In this context, data-driven AI methods, particularly recent advancements in deep learning, have exhibited groundbreaking performance and established themselves as the state of the art for problems in computer vision, natural language processing, and complex decision-making. For example, Mitrokhin et al. describe a hybrid framework for image-based context awareness, training a hash neural network on images to show that *hyperdimensional vectors can be constructed such that vector-symbolic inference arises naturally out of their output.* This enhances the robustness and explainability of the classification process, achieving state-of-the-art accuracy on real-world image datasets such as the popular CIFAR-10.

Acquiring symbol abstractions from raw continuous inputs, i.e., symbol grounding, and decision-making become particularly challenging with the high-dimensional inputs received by robots. Despite the impressive results achieved by deep neural networks and foundation models, their direct use in robots becomes inefficient, hinders transparency, and provides arbitrary responses in novel situations. Hybrid frameworks can address these limitations by leveraging the complementary strengths of symbolic and data-driven AI systems. For example, the framework of Nevens et al. uses symbolic AI to enable an agent to construct *a conceptual system in which meaningful concepts are formed* based on *human-interpretable feature channels.* They use a dataset of images for manipulating blocks to illustrate how concepts acquired from limited data points can be combined and generalized to unseen instances. Sasaki et al. show that grounding robotic gestures *with quantitative meaning calculated from word-distributed representations constructed from a large corpus of text* enable robots to display behavior that humans perceive to be natural. Riley et al. describe a framework that supports non-monotonic logical reasoning with abstractions of prior commonsense knowledge and information extracted by deep neural networks from relevant image regions; they show substantial performance improvement compared with state of the art for visual question answering, and vision-based planning and diagnostics. Furthermore, Grosvenor et al. and Ghiasvand et al. document examples of real-world integration of similar ideas in the context of knowledge-enhanced deep visual tracking of satellites, and a comprehensive architecture for space robotic mission planning and control, respectively.

In summary, the contributions to this topic highlight the importance of merging symbolic and data-driven AI methods in the context of robotics (and AI). These papers demonstrate how such hybrid frameworks enable robots to reason with complex cognitive theories and noisy multimodal sensor observations to achieve reliable, efficient, and transparent scene understanding, planning, diagnostics, and human-robot collaboration in complex simulated and physical domains. The papers also draw attention to the fundamental open problems that need to be addressed to leverage the full potential of robots in practical applications. We hope that these papers will foster further collaboration between the related research communities toward achieving societal benefits.

